# Termites (*Blattodea* Latreille 1810, *Termitoidae* Latreille 1802) of Abuko Nature Reserve, Nyambai Forest Park and Tanji Bird Reserve (The Gambia)

**DOI:** 10.3390/insects10050122

**Published:** 2019-04-28

**Authors:** Abdoulaye Baila Ndiaye, Ebrima Njie, Paul A. Correa

**Affiliations:** 1 Laboratoire de Zoologie des Invertébrés terrestres, IFAN, UCAD, B. P. 206 Dakar, Sénégal; 2 School of Arts and Sciences, Division of Physical and Natural Sciences, University of The Gambia, Brikama Campus, P.O. Box 3530 Serekunda, The Gambia; enjie@utg.edu.gm (E.N.); paul.a.correa@utg.edu.gm (P.A.C.)

**Keywords:** termites, The Gambia, protected site

## Abstract

From 28 October to 5 November 2013, a termite study was undertaken in 3 protected sites in The Gambia (West Africa). The aim of the study is to investigate the diversity of termites in three protected areas in the western region of the country. Termite sampling is carried out in 100 m × 2 m transects that are replicated three (3) times in each site. A total of thirty-one (31) termite species, that belong to fungus growing (11), harvester (1), humuvorous (12) and xylophagous (7), were recorded. The following nineteen (19) species are new to The Gambia: *Coptotermes intermedius*, *Astalotermes* near *quietus*, *Ancistrotermes* *cavithorax*, *Macrotermes* *bellicosus*, *Microtermes* *grassei*, *M. lepidus*, *M. subhyalinus*, *Odontotermes erraticus*, *O. pauperans*, *O.* *sudanensis, Basidentitermes* sp., *Euchilotermes* *tensus* *arcuata*, *Noditermes* *cristifrons*, *Amitermes evuncifer*, *Amitermes spinifer, Microcerotermes fuscotibialis, Microcerotermes* near *parvulus, Microcerotermes* near *solidus and Promirotermes holmgreni.* Additional description and/or ecological information on *Odontotermes* *erraticus*, *Cubitermes* *severus*, *Cubitermes* n. *proximatus*, *Euchilotermes* *tensus* *arcuata*, *Basidentitermes* sp., and *Noditermes* *cristifrons* are given.

## 1. Introduction

The termite fauna of The Gambia is still poorly known. One single termite collection trial carried out by Sands in 1966 in the country has been documented [1].

Prior to this date, only one termite species, *Odontotermes capensis*, referred to as *Termes fatalis*, was wrongly reported to The Gambia by Walker in 1845 [2]. The occurrence of *T*. *fatalis* in both South Africa and The Gambia is objected to by Sjöstedt [3]. The African species referred to as *O. capensis* is restricted to South Africa and does not occur in The Gambia [4].

From 1950 to 2013, thirty (30) termite species (Table 1) were recorded in The Gambia based on the works of Sands [1,5,6,7]; Williams & Perez-Morales [8], Johnson et al. [9] and Krishna et al. [4].

In this study, the objective is to investigate the diversity of termite species and functional groups in the three protected areas of The Gambia, namely Abuko Nature Reserve, Nyambai Forest Park, and Tanji Bird Reserve.

## 2. Material and Methods

### 2.1. Study Sites

**Figure 1:**
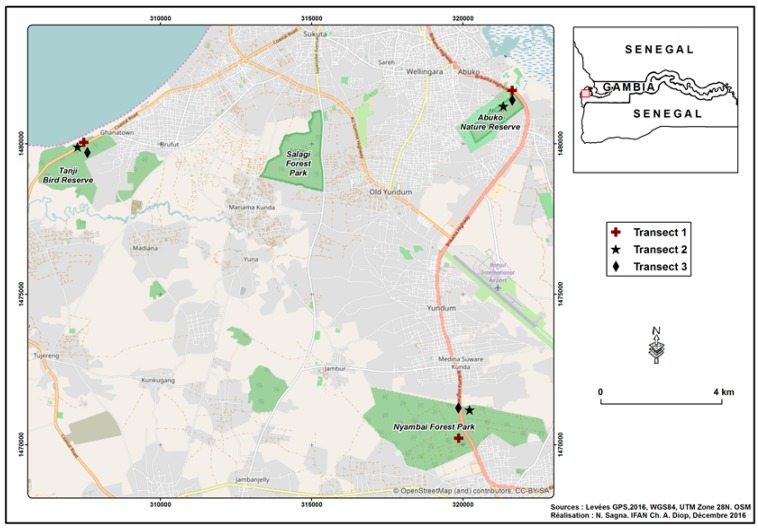
The location of the study sites Abuko Nature Reserve, Tanji Bird Reserve and Nyambai Forest Park.

The Gambia is a small country in West Africa enclosed by the Senegalese territory except on its Atlantic coast (Figure 1). The climate is characterized by a short rainy season from July to September and a dry season during the rest of the year. From the coast to the inland, the rainfall (900–1300 mm) declines and temperatures increase. In the dry season, the inland regions have an average temperature as high as 35 °C, whilst the average temperature in the coastal regions ranges between 25 °C and 28 °C. In the wet season, the average temperature can be below 25 °C at the coast and up to 30 °C in the inland.

The Termites were sampled from the Abuko Nature Reserve, Tanji Bird Reserve and the Nyambai Forest Park, which are protected areas in the coastal region (Figure 1).

### 2.2. Abuko Nature Reserve

The Abuko Nature Reserve is located outside the village of Lamin (13°23′00.45″ N, 16°38′37.9″ W) in the Kombo North District, about 25 km away from Banjul. It has been protected as a water catchment area since 1916 and was officially declared a nature reserve in 1968. With a current size of 106 ha, Abuko Nature Reserve is home to a wide diversity of mammals, birds and invertebrates.

**Figure 2:**
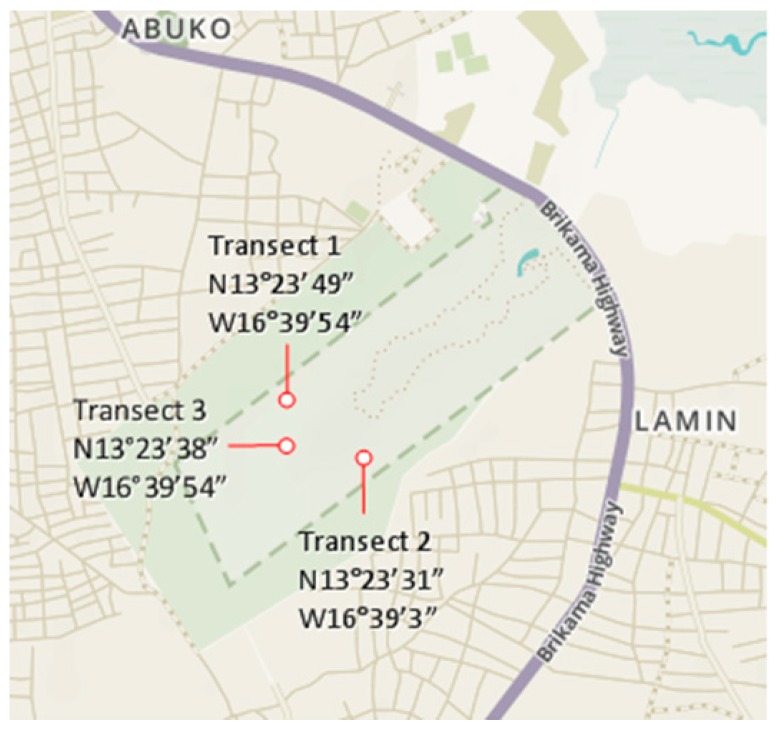
The transects of termite sampling at Abuko Nature Reserve.

Rectangular in shape, the reserve is surrounded by a 300 m wide buffer zone and it is centered on the Lamin village stream which surfaces within the lower half of the reserve, thereby providing a fairly humid microclimate in the heart of the area. The transect locations are shown in Figure 2.

For most of the year, the central part of the reserve is very humid due to the presence of a dense gallery forest, which surrounds a chain of three (3) pools. Soils are sandy in the periphery of the reserve and sandy/muddy towards the center where the tree canopy forms a continuous shade over the lower vegetation particularly during the wet season.

Abuko Nature Reserve is among the least disturbed sites of The Gambia where numerous animal species as well plant species continue to be under strong conservation measures.

### 2.3. Tanji Bird Reserve

**Figure 3:**
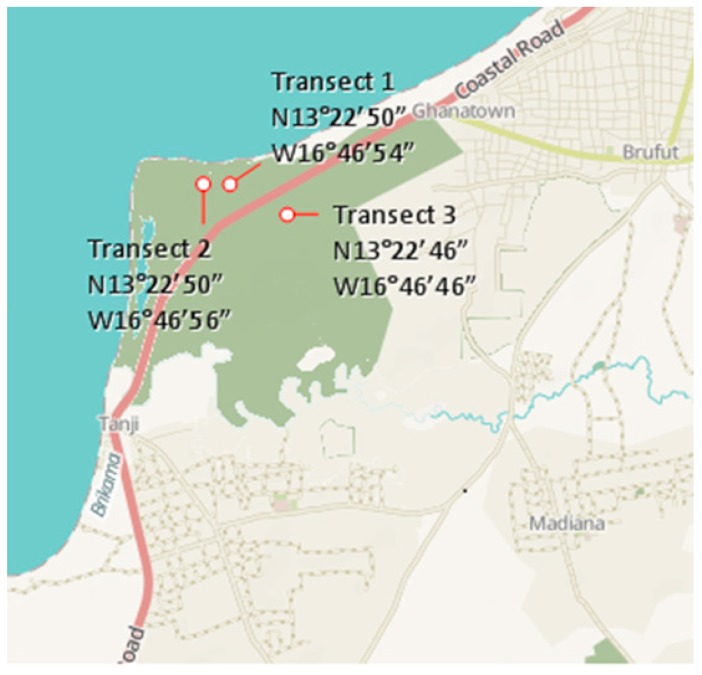
The transect of termite sampling at the Tanji Bird Reserve.

The Tanji River Bird Reserve is located along the Atlantic Coast in the Western Division, Kombo North. It is a few kilometers away from the fishing village of Ghana town (13°23′06.67″ N, 16°46′05.04′’ W). The reserve was established in 1993 and covers a surface area of 612 ha (6.12 km^2^). The three transect locations are shown in Figure 3.

It encompasses the Tanji River and estuary and includes a mangrove ecosystem, coastal dune scrub woodland, and dry woodland savannah. The climate is greatly influenced by the ocean wind.

The northern strip is denser and has a lower canopy height due to previous clearance (transect 1 and transect 2). The southern strip is more open with isolated mature trees as a result of long-term grazing patterns (transect 3). The dominant plant species found are the Ginger Bread Plum, *Parinari macrophylla*, the Rhun Palm, *Borassus aethiopium*, and the Baobab, *Adansonia digitata*. The understorey is generally grass-dominated by the feathery flowered, *Perotis indica*, the stiff leafed *Sporobolus spicatus* and the spiny fruited *Cenchrus biflorus*. A variety of invertebrates populates the reserve, with arthropods being the most abundant.

### 2.4. The Nyambai Forest

**Figure 4:**
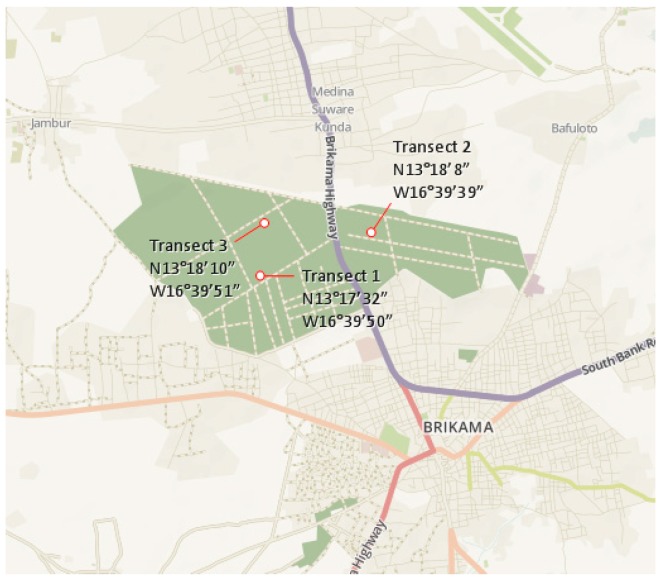
The transects of termite sampling at Nyambai Forest Park.

The Nyambai Forest Park is an artificial forest established in 1964. The park was enriched with the *Gmelina arborea* and *Phyllostachys edullus* species. It is located at midway between Farato village and Brikama (13°16′29.26″ N, 16°38′27.31″ W) about 35 km from Banjul. The transect locations are shown in Figure 4.

The vegetation of the forest park is essentially composed of three different species: a spiny shrub vegetation, a tall but thin *Gmelina arborea* canopy, and a narrow strip of *Phyllostachys* sp. on the northern side.

The soil is completely covered with a thick layer of litter; a few thriving kinds of grasses grow here and there on the finely sandy to muddy soil. The relative humidity is high in the morning and late in the evening. Monthly average of temperatures range between 17–24 °C for the minima and 31–33 °C for the maxima.

### 2.5. Sampling Methods

To standardize sampling effort in the tropical forest areas, Jones & Eggleton [10] developed a protocol based on a 10 m × 2 m transect divided into 20 contiguous sections of 5 m × 2 m. Two experienced people sampled each section for 30 min.

In the studied sites, the sampling method, a derivative of the Jones & Eggleton sampling method, was carried out using a transect (3 transects/site) of 100 m long and 2 m wide. The transect is not subdivided into sections. The duration of the sampling is not limited but depends on the time required to cover the entire transect. In each transect, the termites are sampled by 3 experienced collectors searching for termites in the soil, litter, dead wood, the stump of trees, beneath the bark of trees, and termite arboreal nests.

The distance between transects ranges from 350 to 700 m at Abuko, from 360 to 1150 m at the Nyambai Forest and from 60 to 325 m at Tanji.

The encountered termite soldiers, workers, swarming individuals, kings, and queens are collected and kept in ethanol 70% within labeled containers bearing the name of the site, the date, and the micro-habitat.

The voucher specimens are conserved in the entomological collection of IFAN (University of Cheikh Anta Diop University, Dakar, Senegal). The duplicates of the *Cubitermes* were given to Prof. G. Josens (Université Libre de Bruxelle, Brussels, Belgium) and those of the soldierless termites to Prof. Y. Roisin (Université Libre de Bruxelle, Brussels, Belgium).

### 2.6. Species Identification

Specimens were observed and photographed using a stereomicroscope (Leica M80) equipped with a camera (Leica IC80 HD) connected to a computer. Leica suite application (Las version 4.2.0) is used for image acquisition and mensuration.

Specimens are compared with reference specimens from the IFAN (Institut fondamental d’Afrique noire) collection identified by W. A. Sands. The reference works by Silvestri [11,12], Sjöstedt [3], Emerson [13], Grassé [14,15], Bouillon & Mathot [16] and Roy-Noël [17] are used. The works of Sands focusing more on Nasutitermitinae [18] and on the genus *Amitermes* [19] are also used. Identification of the soldierless species of Apicotermitinae has been made after sands [5,7] on the basis of the morphology of the digestive tube: mesenteron-proctodeum junction and dissected enteric valves are observed under the stereomicroscope. *Cubitermes* species identification is based on the combination of the morphological characters of soldiers [3,11,12,13,17] and the shape of the cushions of workers’ enteric valves [20]. Enteric valves are dissected and mounted between lamellas, then observed under steromicroscope. *Cubitermes* species identenfication has been confirmed by Professor G. Josens (ULB, Belgium) who is working on the revision of the genus.

Measurement procedure of head and mandible.

The head width corresponding to the maximum width in the dorsal view.

The head length is measured in the dorsal view from the occiput to the base of the labrum (soldier) or the anterior of the clypeus (worker).

The length of the left mandible is measured in the dorsal view, from the lateral most proximal visible point to the apical point.

## 3. Results

### 3.1. Termite Diversity in the Three Sites

Thirty-one (31) termite species have been recorded in the three sites. They belong to the following two families, six subfamilies and nineteen genera (Table 2).

### 3.2. New Termite Species Recorded in The Gambia

Among the 31 termite species recorded in Abuko Nature Reserve, Nyambai Forest Park and in Tanji Bird Reserve, 19 termite species are recorded newly from The Gambia.

For both subfamilies, Coptotermitinae (Rhinotermitidae) and Apicotermitinae (Termitidae), one newly recorded species has been found, respectively *Coptotermes intermedius* and *Astalotermes* near *quietus*.

In the Macrotermitinae (Termitidae), the 8 newly recorded species from The Gambia are *Ancistrotermes cavithorax*, *Macrotermes bellicosus*, *Microtermes grassei*, *M. lepidus*, *M. subhyalinus*, *Odontotermes erraticus*, *O. pauperans* and *O. sudanensis*.

The three new species of Cubiterminae (Termitidae) are *Basidentitermes* sp., *Euchilotermes tensus arcuata* and *Noditermes cristifrons*.

The following six species of Termitinae are new to The Gambia: *Amitermes evuncifer*, *Amitermes spinifer*, *Microcerotermes fuscotibialis*, *Microcerotermes near parvulus*, *Microcerotermes near solidus*, *Promirotermes holmgreni*.

### 3.3. Termite Diversity in Abuko Nature Reserve

At the Abuko Nature Reserve, 27 species of termites belonging to 2 families and 5 subfamilies were recorded (Table 3). The variable number of the collected species between transects suggests a certain heterogeneity of the termite distribution in the site.

In terms of functional diversity, there is a predominance of the humivorous termites with 11 species followed by the fungus-growing Macrotermitinae which are represented by 9 species. The xylophagous (6 species) and the haverster termites (1 species) are the least diverse. This type of termite assemblage in Abuko is characteristic of a forestry profile.

### 3.4. Termite Diversity in Nyambai Forest Park

The species richness of termites in Nyambai Forest Park is of 20 species (Table 4). At the functional level, there is still greater diversity of the humivorous termites represented with 12 species followed by the fungus-growing termite (8 species). The harvester termites and the xylophagous termites are represented each by 1 species.

The spatial distribution of the termite species is rather heterogeneous: 12 species are recorded in transect 1, 10 species in transect 2 and 16 species in transect 3. The species richness and spatial distribution heterogeneity in Nyambai Forest are less important than in Abuko and could be associated with the relatively low botanical diversity in this artificial site.

### 3.5. Termite Diversity in Tanji Bird Reserve

At the Tanji Bird Reserve, with 20 species, the species richness is less important than in the other two sites (Table 5). The spatial distribution is also heterogeneous in this site as 15 species are recorded in transect 1, 12 species in transect 2 and 4 species in transect 3.

In terms of functional diversity, the fungus-growing termites (11 species) largely dominate the humivorous (5 species) and the xylophagous (1 species).

### 3.6. Additional Information on Some Species

Based on the frequent confusion and misidentification in the West African *Odontotermes* and *Cubitermes*, we give some descriptive information on *Odontotermes erraticus*, *Cubitermes severus* and *Cubitermes* near *proximatus*. *Euchilotermes tensus arcuata*, a subspecies described by Silvestri, should be elevated to the rank of species, taking into account the distinctive features used in the description of the species of the genus. Finally, some information is given on *Basidentitermes* sp. and *Noditermes cristifrons*.

#### 3.6.1. Odontotermes erraticus Grassé, 1944

**Figure 5:**
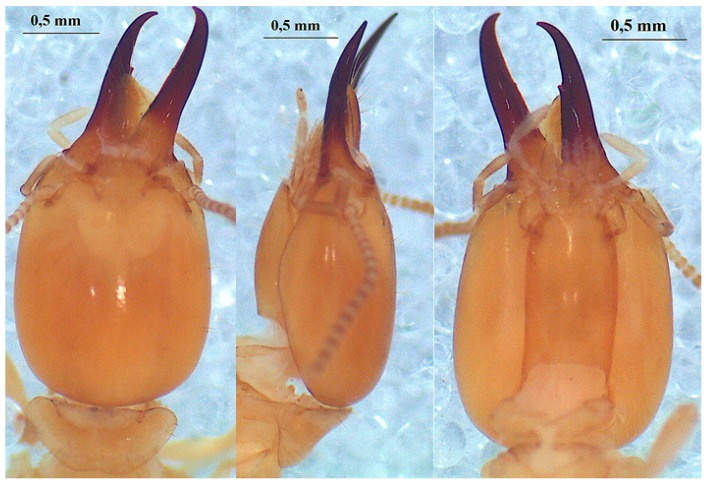
The head of *Odontotermes erraticus* Grassé 1944 soldier in dorsal (left), profile (middle) and ventral (right) views.

The head of the soldier (Figure 5) is yellow-orange in color or dark-brown. The antennae are with 16 antennal segments. The left mandible shows a marginal tooth. The two soldier head measurements are as follows: head length 1.61 mm and 1.64 mm, head width 1.27 mm and 1.28 mm, left mandible length 1.10 mm and 1.15 mm, hind tibia length 1.09 mm.

A large worker has 17 antennal segments in their antennae whereas a small worker individual has 16 antennal segments. Head measurements are shown in Table 6 and Table 7.

#### 3.6.2. *Cubitermes severus* Silvestri, 1914

**Figure 6:**
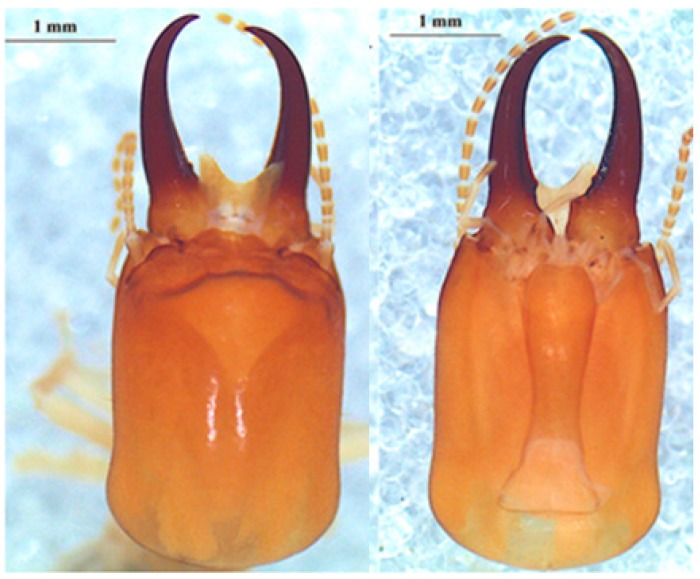
The head of the *Cubitermes severus* Silvestri 1914 soldier in the dorsal (left) and ventral (right) views.

This is a species characterized by the shape (Figure 6) and the size (Table 8) of its soldier. Table 9 shows the dimensions of workers. It is the largest size *Cubitermes* in the collection.

**Figure 7:**
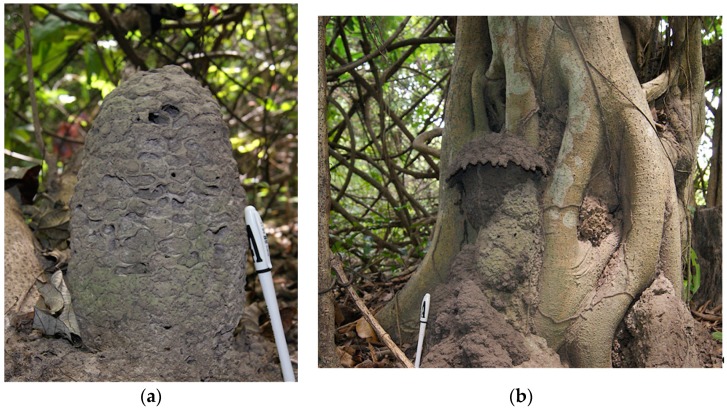
(**a**) The nest of the *Cubitermes severus* Silvestri 1914 without a cap; (**b**) The nest of *Cubitermes severus* Silvestri 1914 with a cap.

*Cubitermes severus* has been collected both in nests without caps (Figure 7a) and in typical mushroom nests (Figure 7b). The column of the nest is much higher than that of *Cubitermes* near *proximatus*. This mound builder species occupies his nest alone or shares it with the inquilines *Promirotermes holmgreni*, *Noditermes cristifrons* and *Pericapritermes urgens*.

#### 3.6.3. *Cubitermes* Near *proximatus* Silvestri, 1914

The observation of the enteric valves of the workers of these *Cubitermes* shows their proximity to *C. proximatus*. However, based on the morphology, the color and the dimensions of the soldier’s head, we divided them into two morphotypes.

##### Morphotype 1 of *Cubitermes* Near *proximatus*

**Figure 8:**
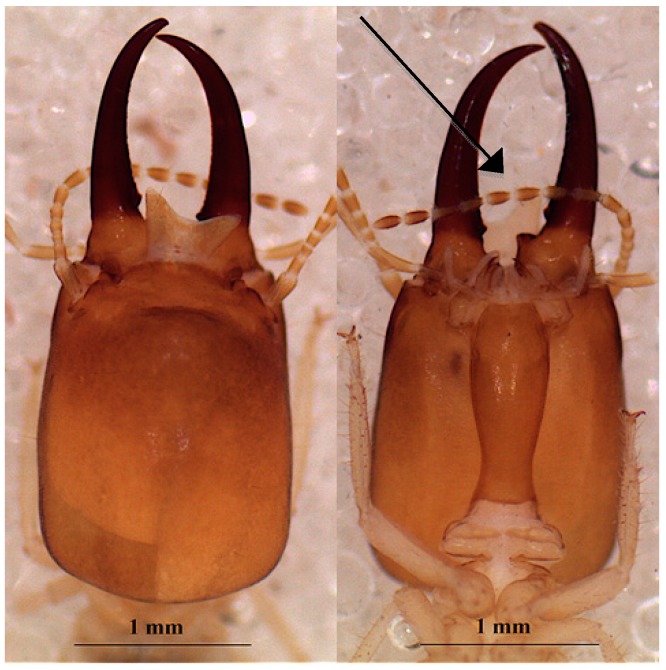
The head of morphotype 1 of *Cubitermes* near a *proximaus* Silvestri 1914 soldier in the dorsal (left) and ventral (right) views (the arrow shows the indentation at the base of the mandible).

This is a species recognizable by the shape and the ochraceus color of the head of its soldiers (Figure 8). Table 10 shows the dimensions of the soldier and Table 11 shows those of the worker.

##### Morphotype 2 of *Cubitermes* Near *proximatus*

**Figure 9:**
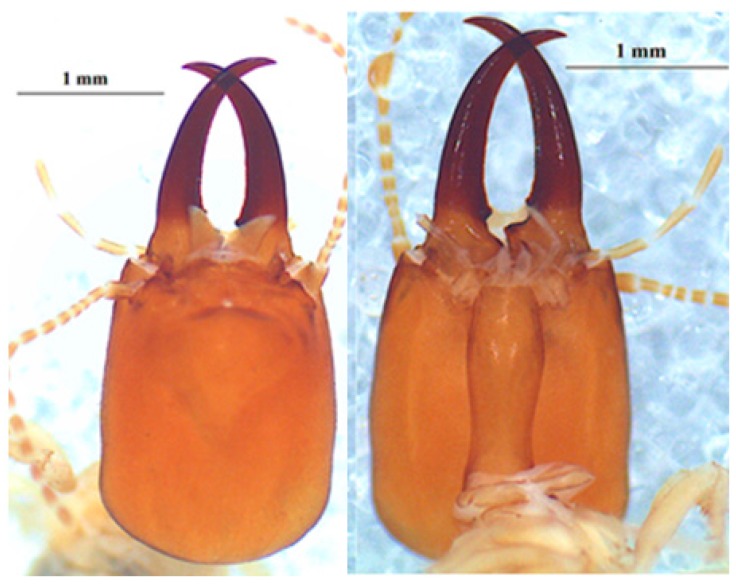
The head of morphotype 2 of *Cubitermes* near the *proximatus* Silvestri 1914 soldier in the dorsal (left) and ventral (right) views.

The soldier of morphotype 2 (Figure 9) is clearly larger (Table 12). Morphologically, differences are noted on the lateral margin of the head, which is less convergent, and the mandibles that are less curved in *C. proximatus.* The indentations at the base of the mandibles (ventral view of Figure 8 and Figure 9) are also distinctive features between the two morphotypes.

The measurements of the morphotype 2 workers are shown in Table 13.

**Figure 10:**
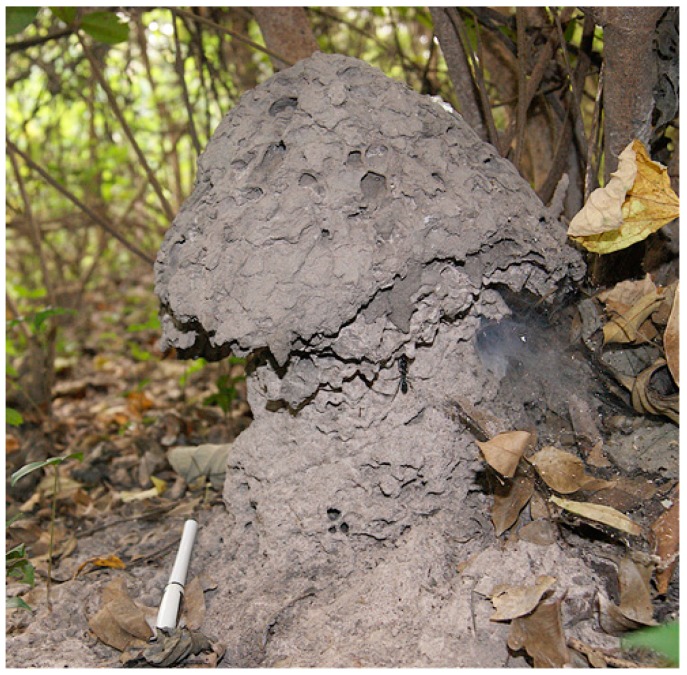
The mushroom nest of *Cubitermes* near *proximatus* Silvestri, 1914.

The mushroom nests of morphotype 2 are small in size (Figure 10). The column is often sufficiently developed to allow a clear distinction with the cap. The nests are occupied solely by the builder or shared with inquilines such as *Allognathotermes hypogeus*, *Euchilotermes tensus arcuata*, *Microtermes* grassei and/or *Promirotermes holmgreni infera*.

#### 3.6.4. Euchilotermes tensus arcuata *Silvestri, 1914*

**Figure 11:**
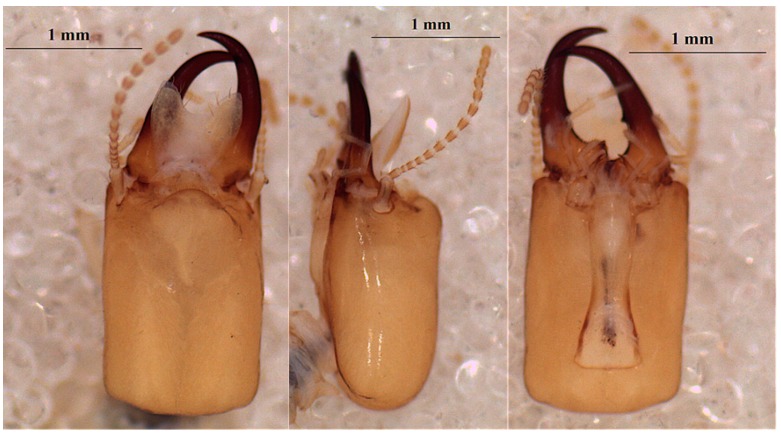
The head of a *Euchilotermes tensus arcuata* Silvestri 1914 soldier in the dorsal (left), profile (middle) and ventral (right) views.

The head of the *Euchilotermes tensus arcuata* soldier (Figure 11) is distinctly rectangular in shape and yellowish in color with light brown mandibles. The mandibles are strongly curved. The labrum is long and wide with two apical large and rounded lobes. The measurements of the soldiers are noted in Table 14 and those of the workers in Table 15.

#### 3.6.5. *Basidentitermes* sp.

The specimens so designated seem different from all known species of the genus. However, more specimens, particularly of soldiers, are needed before the description of a new species.

#### 3.6.6. *Noditermes cristifrons* (Wasmann, 1911)

The measurements of the soldiers of *Noditermes cristifrons* are recorded in Table 16.

**Figure 12:**
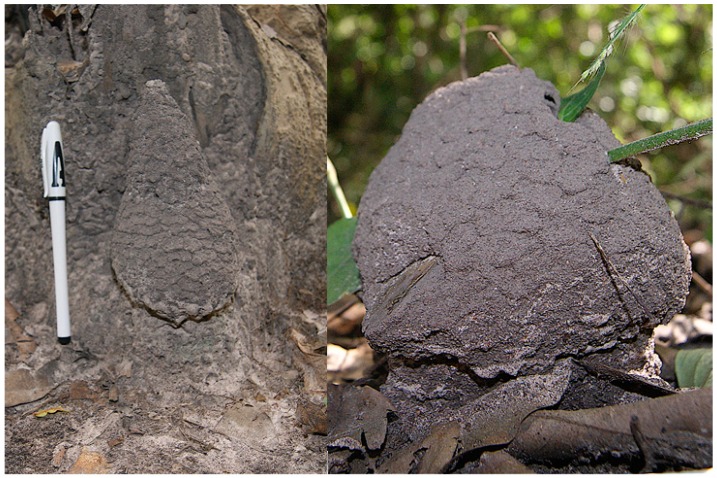
*Noditermes cristifrons:* tree trunk-backed nest (left); freely standing nest (right).

The nests of *Noditermes cristifrons* (Figure 12) are free standing or backed to a tree which affects, in this case, the shape. However, in both cases, the nest displays a scaly appearance. *Noditermes cristifrons* occupies its nest alone or shares it with *Pericapritermes urgens.*

## 4. Discussion

The compilation of the references on the termites of The Gambia gives 30 species for this country. The present study has extended the number of termite species recorded from The Gambia to forty-six (46). Among the thirty-one (31) species that have been newly collected, nineteen (19) species are new for The Gambia and one species among the set is probably new with this field in science.

In Benin, Attignon et al. [21], using the Jones et al. sampling protocol [10], recorded 17 termite species in semi-deciduous forests and 10 species in teak plantations. In a savannah in northern Togo, with the same sampling method, 19 termite species were identified [22].

The protection of sites in The Gambia would explain the greater diversity of their termite fauna. However, in Senegal, in the Kolda region [23] (bordering inner Gambia in the south-east), except for Apicotermitinae, two species of *Basidentitermes*, *Euchilotermes tensus arcuata* and *Amitermes spinifer*, all of the other species recorded in Abuko, Nyambai and Tanji are found.

Of the thirty-one (31) species recorded during this study, only morphotype 2 of *Cubitermes* sp. near *proximatus* and *Euchilotermes arcuata* are not known in Senegal. Morphotype 1 of *Cubitermes* sp. near *proximatus* recorded in Senegal [24,25] was identified as *C. bilobatodes*. *Cubitermes severus* occurs exists in Casamance, Senegal, but has been cited by misidentification as *C. fungifaber* [25].

*Odontotermes erraticus,* described from Niger by Grassé [17], was indeed supposed to be restricted to Niger [4]. Ndiaye [25] points out for the first time its occurrence in Senegal. As one of the newly added species to Gambia’s termite, its presence is seemingly throughout West Africa. *O. erraticus* would be widespread in the Sudano-Sahelian zones of West Africa. Its presence was probably hidden by numerous misidentifications, particularly confusions with the species *O. vulgaris* and *O. latericius* of southern Africa. As Ruelle [26] pointed out, the genus *Odontotermes* is the most complex of the Macrotermitinae.

The genus *Euchilotermes*, exclusively known in the Ethiopian region, comprises four described species [4]. *E. quadriceps* described by Emerson [13] is known in Congo-Zaire (now RD Congo) and Malawi. *E. umbraticola* described by Williams [27,28] is a species of East Africa (Kenya, Tanzania) [4]. Silvestri described *E. tensus* var. *acutidens* and *E. tensus* var. *arcuata* [13]. The variety *acutidens* has been elevated to the rank of species by Emerson [14] on the basis of the following differences: “mandibles more prolonged and curved at apex than with *M. tensus*, the teeth are smaller and sharper with a wider gula”.

According to Krishna et al. [4], *Microtermes hollandei* Grassé is put in synonymy with *M. lepidus* Sjöstedt by Emerson (unpublished catalog). This synonymy is fully justified based on the perfect resemblance between the two species. Grassé [14], the author of the original provisional description of *M. hollandei*, found the differences between the two species as minor and explained them by geographical distribution. However, it should be noted that for both *M. lepidus* [29] and *M. Hollandei* [14], the specimens used in the original description are all from Dakar, Cap vert region, Senegal.

*Microcerotermes fuscotibialis* is easily distinguished by the morphology, the size and the ecology from *M. solidus*, *M. parvus*, and *M. parvulus* which are referred to as small *Microcerotermes* [21]. The difficulties in the discrimination of these small *Microcerotermes* are the source of multiple misidentifications. Described from tropical Africa and cited from all African regions, *M. parvulus* was also recorded from Saudi Arabia [30]. This wide distribution can be explained by the strong plasticity of the species or due to misidentification; the most likely hypothesis. As noted by several authors [24,25,31,32], we believe that the revision of the African *Microcerotermes* is necessary.

## Figures and Tables

**Table 1:** The termite species recorded in The Gambia from 1950 to 2013.

Family	Subfamily	Species	African Distribution
Kalotermitidae Froggatt	Kalotermitinae Froggatt	*Cryptotermes brevis* (Walker, 1853)	RD Congo, The Gambia; Ghana, Madagascar, Nigeria, Senegal, Sierra Leone, South Africa, Uganda
		*Cryptotermes havilandi* (Sjöstedt, 1900)	Cameroon; Congo-Brazzaville; RD Congo, Equatorial Guinea, The Gambia, Ghana, Ivory Coast, Kenya, Madagascar, Mozambique, Namibia, Nigeria, Senegal, Sierra Leone, South Africa, Swaziland, Tanzania, Zimbabwe.
Rhinotermitidae	Coptotermitinae	*Coptotermes sjostedti* Holmgren, 1911	Angola, Cameroon, RD Congo, The Gambia, Ghana, Guinea, Ivory Coast, Mozambique, Nigeria, Senegal, Sierra Leone, Somalia, Sudan, São Tomé and Príncipe, Tanzania, Uganda
Termitidae Latreille	Macrotermitinae Kemner	*Ancistrotermes crucifer* (Sjöstedt, 1897)	Angola, Cameroon, RD Congo, Ethiopia, The Gambia, Ghana, Guinea, Ivory Coast, Nigeria, Senegal, Sierra Leone, Togo
		*Ancistrotermes guineensis* (Silvestri, 1912)	Cameroon, The Gambia, Ghana; Guinea, Guinea-Bissau, Ivory Coast, Nigeria, Senegal
		*Macrotermes subhyalinus* (Rambur, 1842)	Angola, Benin, Burundi, Central African Republic, Chad, RD Congo, Ethiopia, The Gambia, Ghana, Guinea, Guinea-Bissau, Ivory Coast, Kenya, Liberia, Malawi, Mali, Mozambique, Namibia, Nigeria, Rwanda, Senegal, Sierra Leone, Somalia, Sudan, Tanzania, Togo, Uganda, Zambia, Zimbabwe
		*Megaprotermes giffardii* (Silvestri, 1914)	Central African Republic, RD Congo, The Gambia, Ghana, Ivory Coast, Nigeria, Senegal
	Apicotermitinae Grassé & Noirot	*Allognathotermes ivorensis* Grassé and Noirot, 1955	The Gambia, Guinea, Ivory Coast, Nigeria
		*Adaiphrotermes cuniculator* Sands, 1972	The Gambia, Ghana, Nigeria, Senegal
		*Aderitotermes cavator* Sands, 1972	Cameroon, The Gambia; Ivory Coast, Nigeria
		*Alyscotermes kilimandjaricus* (Sjöstedt, 1907)	RD Congo, The Gambia, Guinea, Ivory Coast, Kenya, Malawi, Nigeria, South Africa, Tanzania, Uganda, Zimbabwe
		*Anenteotermes ateuchestes* Sands, 1972	Cameroon, The Gambia
	Nasutitermitinae Hare	*Eutermellus undulans* Sands, 1965	The Gambia, Ghana, Guinea, Nigeria
		*Fulleritermes tenebricus* (Silvestri, 1914)	Central African Republic, RD Congo, Ghana, The Gambia, Guinea, Ivory Coast, Nigeria, Senegal, Sudan
		*Nasutitermes arborum* (Smeathman, 1781)	Central African Republic, RD Congo, Ghana, The Gambia, Guinea, Ivory Coast, Nigeria, Senegal, Sudan
		*Trinervitermes trinervius* (Rambur, 1842)	Central African Republic, Chad, RD Congo, The Gambia, Ghana, Guinea, Guinea-Bissau, Ivory Coast, Liberia, Nigeria, Senegal, South Africa, Uganda
	Cubitermitinae Weidner	*Basidentitermes aurivillii* (Sjöstedt, 1897)	Cameroon, Congo-Brazzaville, RD Congo, The Gambia, Ghana, Nigeria, Sudan, Uganda
		*Basidentitermes potens* Silvestri, 1914	The Gambia, Guinea, Ivory Coast, Nigeria
		*Cubitermes bilobatodes* Silvestri, 1912	The Gambia, Guinea-Bissau, Senegal
		*Cubitermes gaigei* (Emerson, 1928)	Cameroon, Gabon, The Gambia, Guinea, Ivory Coast, Nigeria
		*Cubitermes proximatus* Silvestri, 1914	The Gambia, Guinea
		*Cubitermes severus* Silvestri, 1914	The Gambia, Guinea, Ivory Coast, Nigeria, Sudan
		*Euchilotermes tensus tensus* Silvestri, 1914	Cameroon, The Gambia, Ghana, Guinea, Ivory Coast, Nigeria, Sierra Leone
		*Megagnathotermes notandus* Silvestri, 1914	RD Congo, The Gambia, Guinea, Ivory Coast, Nigeria, Sierra Leone
		*Procubitermes sjostedti* (Rosen, 1912)	The Gambia, Guinea, Ivory Coast, Liberia, Nigeria, Senegal
		*Trapellitermes loxomastax* Sands, 1995	The Gambia, Ghana
	Termitinae Latreille	*Amitermes guineensis* Sands, 1992	The Gambia, Ghana, Nigeria
		*Promirotermes redundans* Silvestri, 1914	The Gambia, Ghana, Guinea, Nigeria, Senegal
		*Pericapritermes nigerianus* Silvestri, 1914	Cameroon, The Gambia, Ghana, Nigeria
		*Pericapritermes* urgens Silvestri, 1914	Cameroon, The Gambia, Ghana, Guinea, Ivory Coast, Nigeria, Senegal

**Table 2:** The termite species recorded in the three protected areas in The Gambia. (ANR: Abuko Nature Reserve; NFP: Nyambai Forest Park; TBR: Tanji Bird Reserve; FG: functional group; FGT: fungus growing termites; Ha: harvester termites; Hu: Humuvorous termites; Xylophagous termites).

Family	Subfamily	Genus	Species	ANR	NFP	TBR	FG
Rhinotermitidae Froggatt, 1897	Coptotermitinae Holmgren, 1910	*Coptotermes* Wasmann, 1896	*Coptotermes intermedius* Silvestri, 1912 *	X			Xyl
Termitidae Latreille, 1802	Macrotermitinae Kemner, 1934	*Ancistrotermes* Silvestri, 1912	*Ancistrotermes cavithorax* (Sjöstedt, 1899) *	X	X	X	FGT
			*Ancistrotermes crucifer* (Sjöstedt, 1897)	X	X	X	
			*Ancistrotermes guineensis* (Silvestri, 1912)	X	X	X	
		*Macrotermes* Holmgren, 1909	*Macrotermes bellicosus* (Smeathman, 1781) *	X	X	X	FGT
			*Macrotermes subhyalinus* (Rambur, 1842)	X		X	
		*Microtermes* Wasmann, 1902	*Microtermes grassei* Ghidini, 1955 *	X	X	X	FGT
			*Microtermes lepidus* Sjöstedt, 1924 *	X	X	X	
			*Microtermes subhyalinus* Silvestri, 1914 *	X		X	
		*Odontotermes* Holmgren, 1910	*Odontotermes erraticus* Grassé, 1944 *			X	FGT
			*Odontotermes pauperans* (Silvestri, 1912) *		X	X	
			*Odontotermes sudanensis* Sjöstedt, 1924 *	X	X	X	
	Apicotermitinae Grassé & Noirot, 1955	*Adaiphrotermes* Sands, 1972	*Adaiphrotermes* near *cuniculator* Sands, 1972	X	X		Hu
		*Aderitotermes* Sands, 1972	*Aderitotermes* near *cavator* Sands, 1972		X		Hu
		*Astalotermes* Sands, 1972	*Astalotermes* near *quietus* Sands, 1972 *	X	X	X	Hu
		*Allognathotermes* Silvestri, 1914	*Allognathotermes ivorensis* Grassé & Noirot, 1954	X			Hu
	Nasutitermitinae Hare, 1937	*Nasutitermes* Dudley, 1890	*Nasutitermes arborum* (Smeathman, 1781)	X			Xyl
		*Trinervitermes* Holmgren, 1912	*Trinervitermes trinervius* (Rambur, 1842)	X	X		Ha
	Cubitermitinae Weidner, 1956	*Basidentitermes* Holmgren, 1912	*Basidentitermes potens* Silvestri, 1914	X	X	X	Hu
			*Basidentitermes* sp. *	X			
		*Cubitermes* Wasmann, 1906	*Cubitermes severus* Silvestri, 1914	X	X	X	Hu
			*Cubitermes* near *proximatus* Silvestri, 1914	X	X	X	
		*Euchilotermes* Silvestri, 1914	*Euchilotermes tensus arcuata* Silvestri, 1914 *	X	X		Hu
		*Noditermes* Sjöstedt, 1924	*Noditermes cristifrons* (Wasmann, 1911) *	X	X	X	Hu
	Termitinae Latreille, 1802	*Amitermes* Silvestri, 1901	*Amitermes evuncifer* (Silvestri, 1912) *	X	X	X	Xyl
			*Amitermes spinifer* (Silvestri, 1914) *			X	
		*Microcerotermes* Silvestri, 1901	*Microcerotermes fuscotibialis* (Sjöstedt, 1896) *	X		X	Xyl
			*Microcerotermes* near *parvulus* (Sjöstedt, 1911) *	X		X	
			*Microcerotermes* near *solidus* Silvestri, 1912 *	X			
		*Pericapritermes* Silvestri, 1914	*Pericapritermes urgens* Silvestri, 1914	X	X		Hu
		*Promirotermes* Silvestri, 1914	*Promirotermes holmgreni* (Silvestri, 1912) *	X	X		Hu

* New records from The Gambia.

**Table 3:** The termite species collected in different stations in the Abuko Nature Reserve. (Abbreviations: K = king; Q = queen; S = soldiers; W = workers).

Abuko Nature Reserve Species	Transect 1	Transect 2	Transect 3
*Coptotermes intermedius* Silvestri, 1912	—	Dead wood in the soil (S, W)	—
*Ancistrotermes cavithorax* (Sjöstedt, 1899)	Litter, dead wood, tree (S, W)	Tree collar (S, W)	Dead wood (S, W)
*Ancistrotermes crucifer* (Sjöstedt, 1897)	Litter, dead wood, tree, soil (S, W)	*Macrotermes* nest wall, dead wood (S, W)	Stump, dead wood, soil (S, W)
*Ancistrotermes guineensis* (Silvestri, 1912)	—	Litter (S, W)	—
*Macrotermes bellicosus* (Smeathman, 1781)	Nest (S, W)	Litter, soil (S, W)	Litter, dead wood (S, W)
*Macrotermes subhyalinus* (Rambur, 1842)	Dead wood (S, W)	Litter, dead wood, soil (S, W)	—
*Microtermes grassei* Ghidini, 1955	Dead wood, soil (S, W)	Nest, litter, dead wood (S, W)	—
*Microtermes lepidus* Sjöstedt, 1924	Litter, dead wood, soil, tree, nest (S, W)	—	—
*Microtermes subhyalinus* Silvestri, 1914	Dead wood, soil (S, W)	Dead wood (S, W)	—
*Odontotermes sudanensis* Sjöstedt, 1924	—	—	Dead palm tree stem (S, W)
*Adaiphrotermes* near *cavator*	Soil (W)	—	Nest (W)
*Astalotermes* near *quietus*	Soil (W)	—	Litter (W)
*Allognathotermes ivorensis* Grassé & Noirot, 1954	Nest (S, W)	—	—
*Nasutitermes arborum* (Smeathman, 1781)	—	—	Dead wood, tree (S, W)
*Trinervitermes trinervius* (Rambur, 1842)	—	—	Nest (S, W)
*Basidentitermes potens* Silvestri, 1914	Nest (S, W)	Soil (S, W)	—
*Basidentitermes* sp.	Soil (S, W)	—	—
*Cubitermes* near *proximatus* Silvestri, 1914	Nest, Soil (S, W)	Nest (S, W)	—
*Cubitermes severus* Silvestri, 1914	—	Nest (S, W)	Nest (S, W)
*Euchilotermes tensus arcuata* Silvestri, 1914	Nest (S, W)	—	—
*Noditermes cristifrons* (Wasmann, 1911)	Nest (S, W)	Nest (K, Q, S, W)	Nest (S, W)
*Amitermes evuncifer* (Silvestri, 1912)	Dead wood (S, W)	—	—
*Microcerotermes fuscotibialis* (Sjöstedt, 1896)	Tree, arboreal nest (S, W)	Trees (S, W)	—
*Microcerotermes* near *parvulus* (Sjöstedt, 1911)	—	—	Tree stump (S, W)
*Microcerotermes* near *solidus* Silvestri, 1912	Nest (S, W)	Dead wood (S, W)	—
*Pericapritermes urgens* Silvestri, 1914	Nest (S, W)	—	Nest (S, W)
*Promirotermes holmgreni* (Silvestri, 1912)	—	Nest (S, W)	Stump, dead wood, nest (S, W)

**Table 4:** The termite species collected in different stations in Nyambai Forest Park (abbreviations: K = king; Q = queen; S = soldiers; W = workers).

Nyambai Forest Park Species	Transect 1	Transect 2	Transect 3
*Ancistrotermes cavithorax* (Sjöstedt, 1899)	Tree, dead wood, litter (S, W)	Dead wood (S, W)	Tree, dead wood (S, W)
*Ancistrotermes crucifer* (Sjöstedt, 1897)	Tree (S, W)	—	Stump, tree (S, W)
*Ancistrotermes guineensis* (Silvestri, 1912)	Tree, dead wood (S, W)	Tree, dead wood, liana, soil (S, W)	Tree, dead wood, litter, soil (S, W)
*Macrotermes bellicosus* (Smeathman, 1781)	Nest, dead wood, litter (S, W)	—	Nest, stumps, tree, litter, dead wood, soil (S, W)
*Microtermes grassei* Ghidini, 1955	—	—	Nest (S, W)
*Microtermes lepidus* Sjöstedt, 1924	Tree, dead wood (S, W)	—	Dead wood, soil, nest (S, W)
*Odontotermes pauperans* (Silvestri, 1912)	—	—	Dead wood, soil (S, W)
*Odontotermes sudanensis* Sjöstedt, 1924	Tree, litter, dead wood (S, W)	—	Nest, stump (S, W)
*Adaiphrotermes* near *cuniculator*	—	—	Nest, soil (W)
*Aderitotermes* near *cavator*	Soil (W)	Nest (W)	Runways on tree, soil, nest (W)
*Astalotermes* near *quietus*	—	Soil (W)	Soil (W)
*Trinervitermes trinervius* (Rambur, 1842)	Soil (S, W)	—	—
*Basidentitermes potens* Silvestri, 1914	—	Soil (S, W)	—
*Cubitermes severus* Silvestri, 1914	Nest (S, W)	Nest (Q, S, W)	Nest (S, W)
*Cubitermes* near *proximatus* Silvestri, 1914	Nest (S, W)	—	Nest (S, W)
*Euchilotermes tensus arcuata* Silvestri, 1914	—	—	Nest (S, W)
*Noditermes cristifrons* (Wasmann, 1911)	Soil (S, W)	Nest (K, Q, S, W)	Nest (S, W)
*Amitermes evuncifer* (Silvestri, 1912)	Dead wood (S, W)	Shrub (S, W)	—
*Pericapritermes urgens* Silvestri, 1914	—	Nest (S, W)	Nest (S, W)
*Promirotermes holmgreni* (Silvestri, 1912)	—	Nest (S, W)	—

**Table 5:** The termite species collected in different stations in the Tanji Bird Reserve (abbreviations: S = soldiers; W = workers).

Tanji Bird Reserve Species	Transect 1	Transect 2	Transect 3
*Ancistrotermes cavithorax* (Sjöstedt, 1899)	Stump, soil (S, W)	Dead wood (S, W)	Nest, dead wood (S, W)
*Ancistrotermes crucifer* (Sjöstedt, 1897)	Dead wood (S, W)	—	—
*Ancistrotermes guineensis* (Silvestri, 1912)	Dead wood, soil (S, W)	Litter, dead wood, tree (S, W)	—
*Macrotermes bellicosus* (Smeathman, 1781)	Tree, soil (S, W)	Litter, soil (S, W)	Nest, dead wood (S, W)
*Macrotermes subhyalinus* (Rambur, 1842)	Dead wood in soil (S, W)	—	—
*Microtermes grassei* Ghidini, 1955	Soil (S, W)	—	—
*Microtermes lepidus* Sjöstedt, 1924	Dead wood (S, W)	—	—
*Microtermes subhyalinus* Silvestri, 1914	Dead wood (S, W)	Dead wood, soil (S, W)	—
*Odontotermes erraticus* Grassé, 1944	—	Preys of *Megaponera*, soil (S, W)	—
*Odontotermes pauperans* (Silvestri, 1912)	—	Nest, dead wood (S, W)	Soil (S, W)
*Odontotermes sudanensis* Sjöstedt, 1924	—	Nest, soil (S, W)	—
*Astalotermes* near *quietus*	Soil, Nest (Pseudoecy), runway on tree (W)	—	—
*Basidentitermes potens* Silvestri, 1914	Nest, soil (S, W)	—	—
*Cubitermes* near *proximatus* Silvestri, 1914	Nest, soil (S, W)	—	—
*Cubitermes severus* Silvestri, 1914	—	Nest (S, W)	—
*Noditermes cristifrons* (Wasmann, 1911)	Nest (S, W)	Nest (S, W)	Nest, soil (S, W)
*Amitermes evuncifer* (Silvestri, 1912)	Nest, stump (S, W)	Stump, dead wood (S, W)	—
*Amitermes spinifer* (Silvestri, 1914)	—	Nest (S, W)	—
*Microcerotermes fuscotibialis* (Sjöstedt, 1896)	Tree (S, W)	Tree (S, W)	—
*Microcerotermes* near *parvulus* (Sjöstedt, 1911)	Nest (S, W)	—	—

**Table 6:** The measurements (mm) of the large workers of *Odontotermes erraticus* Grassé.

Worker	Range	Mean	Number
Head length	1.25–1.34	1.29	5
Head width	1.33–1.40	1.36	5
Hind tibia length	1.05–1.11	1.08	4

**Table 7:** The measurements (mm) of the small workers of *Odontotermes erraticus* Grassé.

Worker	Range	Mean	Number
Head length	0.85–0.87	0.86	3
Head width	0.91–0.92	0.91	3
Hind tibia length	0.86–0.88	0.87	3

**Table 8:** The measurements (mm) of the soldiers of *Cubitermes severus* Silvestri, 1914.

Soldier	Range	Mean	Number
Head length	2.87–3.08	2.99	9
Head width	1.96–2.11	2.03	9
Length of left mandible	1.98–2.03	2.01	9
Hind tibia length	1.57–1.69	1.63	8

**Table 9:** The size measurements (mm) of the workers of *Cubitermes severus* Silvestri, 1914.

Worker	Range	Mean	Number
Head length	1.11–1.19	1.14	5
Head width	1.19–1.24	1.21	5
Hind tibia length	1.25	1.29–1.27	4

**Table 10:** The measurements (mm) of the soldiers of morphotye 1 of *Cubitermes* near *proximatus* Silvestri, 1914.

Soldier	Range	Mean	Number
Head length	1.76–1.98	1.89	8
Head width	1.37–1.49	1.43	8
Length of the left mandible	1.44–1.50	1.48	8
Hind tibia length	1.14–1.16	1.14	4

**Table 11:** The measurements (mm) of the workers of morphotype 1 of *Cubitermes* near *proximatus* Silvestri, 1914.

Worker	Range	Mean	Number
Head length	0.82–0.89	0.85	4
Head width	0.93–0.94	0.93	4
Hind tibia length	0.92–0.95	0.94	3

**Table 12:** The measurements (mm) of the soldiers of morphotype 2 of *Cubitermes* near *proximatus* Silvestri, 1914.

Soldier	Range	Mean	Number
Head length	2.05–2.19	2.13	8
Head width	1.50–1.65	1.59	8
Length of left mandible	1.60–1.70	1.64	8
Hind tibia length	1.24–1.32	1.28	7

**Table 13:** The measurements (mm) of the workers of morphotype 2 *Cubitermes* near *proximatus* Silvestri, 1914.

Worker	Range	Mean	Number
Head length	0.96–1.04	1.04	7
Head width	1.01–1.05	1.03	7
Hind tibia length	0.98–1.01	1.00	7

**Table 14:** The measurements (mm) of the soldiers of *Euchilotermes tensus arcuata* Silvestri, 1914.

Soldier	Range	Mean	Number
Head length	1.76–1.85	1.82	6
Head width	1.16–1.24	1.21	6
Length of left mandible	1.15–1.19	1.17	6
Hind tibia length	0.89–0.97	0.91	6

**Table 15:** The measurements (mm) of the workers of *Euchilotermes tensus arcuata* Silvestri, 1914.

Worker Individual	Range	Mean	Number
Head length	0.75–0.84	0.79	4
Head width	0.81–0.88	0.84	4
Hind tibia length	0.77–0.84	0.81	4

**Table 16:** The measurements (mm) of the soldiers of *Noditermes cristifrons* (Wasm.).

Soldier	Range	Mean	Number
Head length	1.16–1.32	1.24	15
Head width	1.00–1.10	1.04	15
Length of left mandible	1.30–1.46	1.38	15
Hind tibia length	0.76–0.82	0.80	8

## References

[1] Sands W.A. (1968). New species and records of Nasutitermitinae (Isoptera: Termitidae) from Africa. Proc. R. Entomol. Soc. Lond..

[2] Snyder T.E. (1949). Catalog of the termites (Isoptera) of the World. Smithsonian Miscellaneous Collections.

[3] Sjöstedt Y. (1925). Revision der Termiten Afrikas. 3. Monographie. Kungl Svenska Vetenska Akademiens Handlingar.

[4] Krishna K., Grimaldi D.A., Krishna V., Engel M.S. (2013). Treatise on the Isoptera of the World. 4. Termitidae (part one). Bulletin of the American Museum of Natural History.

[5] Sands W.A. (1972). The soldierless termites of Africa (*Isoptera*, Termitidae). Bulletin of the British Museum (Natural History) Entomology, Supplement.

[6] Sands W.A. (1995). New genera and species of soil feeding termites (Isoptera: Termitidae) from African savannas. J. Nat. Hist..

[7] Sands W.A. (1998). The Identification of Workers Castes of Termites Genera from Soils of Africa and the Middle East.

[8] Williams R.M.C., Perez-Morales J.V., Jaisson P. (1983). The effect of group size on the survival and feeding economy of pseudoworkers of building damaging *Cryptotermes* spp. (lsoptera, Kalotermitidae). Social Insects in the Tropics.

[9] Johnson R.A., Lamb R.W., Sands W.A., Shittu M.O., Williams R.M.C., Wood T.G. (1980). A check list of Nigerian termites (Isoptera) with brief notes on their biology and distribution. Niger. Field.

[10] Jones D.T., Eggleton P. (2000). Sampling termite assemblages in tropical forests: Testing a rapid biodiversity assessment protocol. J. Appl. Ecol..

[11] Silvestri F. (1912). Termitidi raccolti da L. Fea alla Guinea Portoghese e alla Isole, S. Thomé, Annobon, Principe e Fernando Poo. Annali Museo Civico di Storia Naturale di Genova.

[12] Silvestri F. (1914). Contribuzione alla conoscenza dei Termitidi e Termitophili dell’Africa occidentale. I. Termitidi. Bolletino del Laboratorio di Zoologia Generale e Agraria della R Scuola Superiore d’Agricoltura.

[13] Emerson A.E. (1928). Termites of the Belgian Congo and the Cameroon. Bull. Am. Mus. Nat. Hist..

[14] Grassé P.P. (1937). Recherches sur la systématique et la biologie des termites de l’Afrique occidentale française. Première partie: Protermitidae, Mesotermitidae et Metatermitidae (Termitinae). Annales de la Société Entomologique de France.

[15] Grassé P.P. (1944). Recherches sur la biologie des termites champignonnistes (Macrotermitinae). Annales des Sciences Naturelles.

[16] Bouillon A., Mathot G. (1965). Quel est ce termite africain?. Zooleo.

[17] Roy-Noël J. (1969). Le parc national du Niokolo-Koba. VIII. Isoptera. Mémoire de l’IFAN.

[18] Sands W.A. (1965). A revision of the Termites subfamily Nasutitermitinae (Isoptera): Termitidae from the Ethiopian region. Bulletin of the British Museum (Natural History) Entomology, Supplement 4.

[19] Sands W.A. (1992). The Termites Genus *Amitermes* in Africa and the Middle. Nat. Resour. Inst. Bull..

[20] Josens G. (2008). Bilingual key of the West African Cubitermes soldiers and workers. Taxonomy of West African Termites: Challenges and Prospects.

[21] Attignon S.E., Lachat T., Sinsin B., Nagel P., Paveling R. (2005). Termite assemblages in a West-African semi-deciduous forest and teak plantations. Agric. Ecosyst. Environ..

[22] Schyra J., Korb J. (2019). Termites Communities along a Disturbance Gradient in a West African Savanna. Insects.

[23] Sane H., Samb T., Ndiaye A.B., Ba C.T. (2016). Etude De La Diversite Des Termites (*Isoptera*) Dans Quelques Localites De La Region De Kolda (Haute Casamance, Senegal). Eur. Sci. J..

[24] Roy-Noël J. (1974). Recherches sur l’écologie des Isoptères de la presqu’île du Cap-Vert (Sénégal). Bulletin IFAN Série A.

[25] Ndiaye A.B. (2014). Contribution à la Connaissance des Termites (Isoptera Brullé, 1832) du Sénégal: Systématique et Écologie. Partie, I. Systématique. Ph.D. Thesis.

[26] Ruelle J.E., Werger M.J.A., van Bruggen A.C., Dr W., Junk B.V. (1978). Isoptera. Biogeography and Ecology of Southern Africa.

[27] Williams R.M.C. (1954). New Est African Termitinae (Isoptera: Termitidae). Proc. R. Entomol. Soc. Lond. B.

[28] Williams R.M.C. (1962). A correction Concerning two East African Termitinae (Isoptera: Termitidae). Proc. R. Entomol. Soc. Lond. B.

[29] Sjöstedt Y. (1924). Weitere Neuheiten von der afrikanischen Termitenfauna. Rev. Zool. Afr..

[30] Faragalla R.A.A., Al Qhtani M.H. (2013). The Urban termite fauna (*Isoptera*) of Jeddah City, Western Saudi Arabia. Life Sci. J..

[31] Noirot C. (1955). Termites du centre et du sud-ouest de l’Angola récoltés par A. de Barros Machado. Publiçaões Culturais de Companhia Diamantes de Angolas.

[32] Josens G. (1972). Etudes Biologique et Écologique des Termites (Isoptera) de la Savane de Lamto-Pakobo (Côte d’Ivoire). Ph.D. Thesis.

